# Review article: dietary fibre–microbiota interactions

**DOI:** 10.1111/apt.13248

**Published:** 2015-05-24

**Authors:** H. L. Simpson, B. J. Campbell

**Affiliations:** ^1^ Department of GastroenterologyInstitute of Translational MedicineUniversity of LiverpoolLiverpoolUK

## Abstract

**Background:**

Application of modern rapid DNA sequencing technology has transformed our understanding of the gut microbiota. Diet, in particular plant‐based fibre, appears critical in influencing the composition and metabolic activity of the microbiome, determining levels of short‐chain fatty acids (SCFAs) important for intestinal health.

**Aim:**

To assess current epidemiological, experimental and clinical evidence of how long‐term and short‐term alterations in dietary fibre intake impact on the microbiome and metabolome.

**Methods:**

A Medline search including items ‘intestinal microbiota’, ‘nutrition’, ‘diet’, ‘dietary fibre’, ‘SCFAs’ and ‘prebiotic effect’ was performed.

**Results:**

Studies found evidence of fibre‐influenced differences in the microbiome and metabolome as a consequence of habitual diet, and of long‐term or short‐term intervention (in both animals and humans).

**Conclusions:**

Agrarian diets high in fruit/legume fibre are associated with greater microbial diversity and a predominance of *Prevotella* over *Bacteroides*. ‘Western’‐style diets, high in fat/sugar, low in fibre, decrease beneficial Firmicutes that metabolise dietary plant‐derived polysaccharides to SCFAs and increase mucosa‐associated Proteobacteria (including enteric pathogens). Short‐term diets can also have major effects, particularly those exclusively animal‐based, and those high‐protein, low‐fermentable carbohydrate/fibre ‘weight‐loss’ diets, increasing the abundance of *Bacteroides* and lowering Firmicutes, with long‐term adherence to such diets likely increasing risk of colonic disease. Interventions to prevent intestinal inflammation may be achieved with fermentable prebiotic fibres that enhance beneficial Bifidobacteria or with soluble fibres that block bacterial–epithelial adherence (contrabiotics). These mechanisms may explain many of the differences in microbiota associated with long‐term ingestion of a diet rich in fruit and vegetable fibre.

## Introduction

The human gut contains a dense and diverse microbial community (microbiota) and the application of affordable, modern rapid high‐throughput nucleic acid sequencing technologies has transformed our understanding of its dynamic complexity.^1^, ^2^ Current available metagenomic, metatranscriptomic, metaproteomic and (meta)metabolomic approaches (Table 1) and complementary bioinformatics/computational meta'omic modelling tools can now accurately characterise (albeit with some limitations) compositional changes and function/activity profiles of key microbial communities, and their interactions with the gut environment and with the host.^3^, ^4^, ^5^, ^6^, ^7^, ^8^

**Table 1:** Advanced, high‐throughput approaches used to study variations in the gut microbiome

High‐throughput microbiome sequencing technology	Microbial material	Characteristics/advantages	Limitations	Applications
16S rRNA gene/16S rDNA amplicon analysis (e.g. 454 pyrosequencing, Illumina MiSeq)	gDNA	Fast, cheap sequencing Survey of large communities Revealing bacterial diversity Detecting dysbiosis	Amplification bias Taxonomic information only Comparison of results requires amplification of same region	Microbial composition dysbiosis Identifying healthy and disease‐specific genera/species
Whole genome shotgun metagenomics	gDNA	High coverage, deep sequencing of the total genes present No amplification bias like 16S Uncovering microbial diversity Finding novel genes Bioinformatic screening of host sequences	Expensive Requires high‐depth coverage Assembly of metagenomes complicated due to uneven coverage Bioinformatic analyses complex/time‐consuming No microbial expressed functions	Microbial composition dysbiosis Finding disease‐specific genes Identifying functional‐based studies
Metatranscriptomics	mRNA	Obtaining gene expression profiling Revealing different microbial gene expression across health, disease and different treatment conditions	Instability of mRNA Multiple purification steps needed Lack of reference databases No unique protocol Isolated and transient picture of a diverse and complex community	Revealing functional dysbiosis Enrichment of metagenomic data View of transcriptionally active/functional subset of the genes under investigation
Metaproteomics	Proteins	Obtaining dynamic microbiota protein profiles Comparing microbial patterns across different health, disease and treatment conditions	Technologically challenging Hard to extract total protein (interfering compounds and membrane/matrix‐bound proteins) No unique protocol Bioinformatic analyses of protein mass or sequences is complex/time‐consuming	Confirming microbial function Identifying eukaryotic–prokaryotic analogues To verify metagenomic and metatranscriptomic data Protein inference – finding protein coding, functional sequences and potential roles
Metabolomics	Metabolites	Obtaining metabolic profiles Comparing metabolomes across different disease and treatment conditions	Differentiating host vs. microbial metabolite profiles Lack of reference databases No unique protocol	Identifying and confirming new microbiota and host metabolic pathways/responses Novel biomarker discovery

Initiatives such as MetaHIT (http://www.metahit.eu/) and the Human Microbiome Project (http://hmpdacc.org/) have described the composition and molecular functional profile of intestinal microbiome. On average, the healthy (normal) adult human gut microbiota consists of 10^13^–10^14^ micro‐organisms, with the collective genome of the microbiota (‘microbiome’) estimated to contain 150 times as many genes than that of our own human genome^9^, ^10^ with over 1000 prevalent species identified with a typical individual carrying about 160 species.^10^ The intestinal microbiota plays an important role in key nutritional,^11^ metabolic^11^ and immunological processes.^12^ It is therefore not surprising that perturbations in its composition have been implicated in many diseases and disorders, including inflammatory bowel disease (IBD), obesity and diabetes.^13^, ^14^, ^15^

The intestinal microbiota becomes established in stages through early life, which begins antenatally.^16^, ^17^ Interestingly, initial bacterial colonisers of the gut are largely determined by the mode of delivery; infants born naturally are initially inoculated by bacteria typically present in the vaginal and faecal microbiota, such as *Lactobacillus* and *Prevotella* spp., while those born by caesarean section are colonised by bacteria from the skin and environment.^18^ Indeed, the most significant change in microbiota composition occurs during weaning with introduction to solid foods resulting in a shift within the early 2–3 years of life towards an adult microbiota.^19^, ^20^ Once established, the microbiota remains remarkably stable over time, although it has been suggested that decreased stability and altered diversity of the gut microbiota occurs with changes in body mass index (BMI)^21^ and advancing age.^22^

In healthy adults, although the intestinal microbiota consists of several hundred bacterial species with significant inter‐individual differences, over 90% present belong to the Firmicutes and Bacteroidetes, with the relative abundance of these two major phyla remaining relatively stable in health, albeit with noted large inter‐individual differences in Firmicutes/Bacteroidetes ratio.^10^ Certain bacterial species are also consistently present in most individuals, indicating perhaps presence of a core microbiome.^23^, ^24^, ^25^, ^26^ Large‐scale sequence analysis had suggested that the microbial composition of all individuals, independent of their ethnicity, sex, age or body weight, might exist within three distinctive ‘enterotype’ clusters, predominated by *Bacteroides*,* Prevotella* or *Ruminococcus* spp.^27^ However, it has recently been acknowledged that *Bacteroides* and *Ruminococcus* tend to vary continuously between and within these putative ‘enterotypes’, challenging whether these discrete clusters are actually present and even if potential enterotype‐disease associations exist, particularly given the substantial shifts observed in the microbiome in intestinal inflammation and disease. Similar intra‐‘enterotype’ variation has also been noted for *Prevotella*, and even completely absent from the microbiome in some elements of the population.^28^, ^29^, ^30^

While clearly the intestinal microbiota does remain stable over time, it can be significantly affected by a number of host and environmental/external factors including host genotype^26^ and immunological response,^31^ antibiotic usage,^32^ diet,^20^, ^33^ and exercise.^34^ Dietary composition, modification and interventions in particular have marked impact on gut microbiota diversity, understandable given that resident micro‐organisms obtain energy for growth via metabolism of dietary nutrients and the intermediate and end products of dietary fibre fermentation.^35^

Consumption of dietary fibre significantly alters the composition of the intestinal microbiota.^36^ Hence, a greater understanding of the interaction between dietary fibre and the intestinal microbiota could represent a means of maintaining or improving the microbiota, particularly when dysbiosis exists. The aim of this review was to examine in detail the long‐term and short‐term impact of dietary fibre (and its various components, plant‐derived polysaccharides) on the intestinal microbiota, particularly with respect to its effect on, (i) the composition of the intestinal microbiota, (ii) its role in generating short‐chain fatty acids (SCFAs) – the end products of fermentation of dietary carbohydrate/fibre and energy source for the intestinal epithelium and (iii) in the context of intestinal bacteria–epithelial interactions.

## Search strategy and selection criteria

We searched PubMed using the term ‘intestinal microbiota’ in combination with ‘nutrition’, ‘diet’, ‘dietary fibre’ and ‘short chain fatty acid or SCFA’, and also ‘dietary fibre’ in combination with ‘prebiotic effect’. Publications obtained (from 1968 to 30 November 2014) were reviewed, with emphasis placed particularly, but not exclusively, on high‐quality peer‐reviewed research papers and review articles published in the last 10 years. Reference lists of articles identified by this search strategy were also reviewed and our bibliography was also modified on the basis of comments from peer reviewers to ensure significant publications were not missed, and inclusion of recent articles published late 2014/early 2015.

Searches on ‘probiotics’ and ‘intestinal microbiota’ were not conducted as part of this review, but there is a significant body of evidence indicating that ingestion of probiotic beneficial bacteria likely impacts (albeit transiently) on both composition and metabolism of the intestinal microbiome.^37^, ^38^, ^39^

## Impact of dietary fibre on the intestinal microbiota

Dietary fibre of edible plants comprises insoluble and soluble carbohydrates including cellulose, lignin, and nonstarch polysaccharides (NSP) such as hemicelluloses, pectins and arabinoxylan oligosaccharides.^40^ Other dietary fibre components include nondigestible oligosaccharides such as inulin and oligofructose, as well as resistant starch (RS).^40^, ^41^ They demonstrate resistance to digestion in the human small intestine, allowing passage largely intact into the colon where they increase viscosity and bulking of the faecal matter.^36^ Importantly, it is here that dietary fibre undergoes fermentation by the resident anaerobic colonic microbiota to SCFAs (primarily butyrate, acetate and propionate) that act as the primary carbon energy source for colonocytes.^42^, ^43^, ^44^ There is significant association between levels of SCFAs and composition of the microbiota, with high luminal concentrations resultant of fermentation lowering colonic pH (5.5–6.5 in proximal colon where fermentation is highest, compared to pH 6.5–7.0 in the distal colon) and inhibit growth of Gram‐negative Enterobacteriaceae including familiar pathogens *Salmonella* spp. and *Escherichia coli*.^45^, ^46^ In particular, butyrate has been reported to be protective against development of colitis^47^ and colorectal cancer.^48^, ^49^ Conflicting this dogmatic belief is a recent study by Belcheva *et al*., which demonstrates that microbial‐derived butyrate may in fact drive colon polyp formation *in vivo*, acting as an oncometabolite.^50^, ^51^ Colorectal instillation of butyrate promoted aberrant proliferation and transformation of cancer‐initiated intestinal epithelial cells of mice bearing both *Apc* ^Min/+^ (adenomatous polyposis coli gene, multiple intestinal neoplasia) and *Msh2* ^−/−^ (MutS homologue 2 mismatch repair gene) mutations. Ingestion of a diet low in fermentable carbohydrate (where 7% of the calories provided derived from carbohydrate, as compared to 58% for a normal diet) resulted in lower abundance of Firmicutes, including *Clostridiaceae*,* Lachnospiraceae* and *Ruminococcaceae* families known to generate butyrate, with concomitant reduction in polyps in the small intestine (~twofold) and colon (~sixfold).^50^ The idea that butyrate may have paradoxical effects is not something new, with differential effects previously observed in normal vs. colorectal tumour cell lines, likely due to the Warburg effect.^51^

### Epidemiological evidence

**Figure 1:**
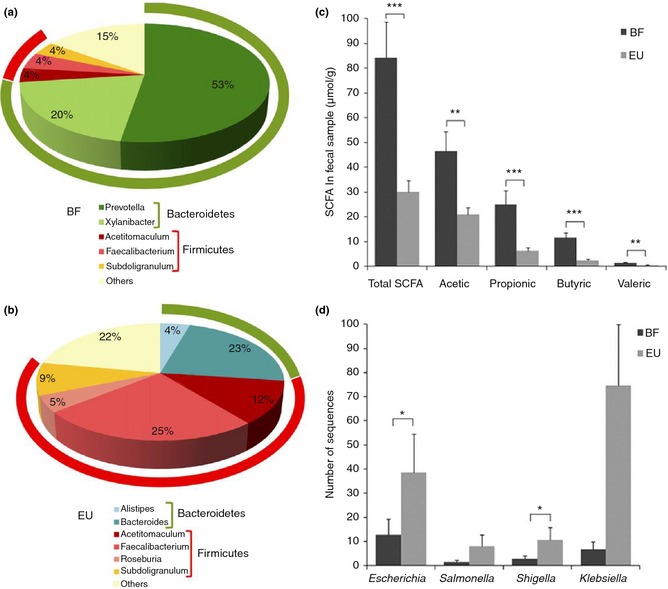
A plant‐based agrarian diet significantly impacts on the diversity of the intestinal microbiota, which subsequently influences the metabolome. 16S rRNA gene analysis reveal a clear separation of bacterial genera present (>3%) in faecal samples of (a) African (Burkino Faso, BF) and (b) European (EU) children. Pie charts are median values. Outer rings represent corresponding phylum (Bacteroidetes, in green; Firmicutes, in red) for each of the most frequently represented genera. (c) SCFAs are higher in faecal samples from BF vs. EU populations as assessed by SPME‐GC‐MS. (d) Principal Enterobacteriaceae (potentially pathogenic intestinal bacteria) identified are lower in abundance in the microbiota of BF children consuming a diet rich in fruit and legume fibre. Mean (±S.E.M.) are plotted. Significant differences, *P <* *0.05; **P ≤* *0.01; ***P ≤* *0.001 (one‐tailed Student's *t‐*test of all data points). De Filippo *et al*. 2010; *Proc Natl Acad Sci USA* 2010; 107(33):14691–6.^33^ Reproduced with permission.

Recent cross‐sectional studies in globally distinct populations suggest that diet significantly impacts on the diversity of the intestinal microbiota, which subsequently influences the metabolome.^33^, ^52^, ^53^, ^54^ The landmark study by De Filippo *et al*.^33^ demonstrated that habitual diet, which typically varies in dietary fibre intake, has distinct long‐term effect on the composition of the intestinal microbiota. The faecal microbiome of healthy children (aged 1–6 years old) living in a rural African village in Burkina Faso, consuming a plant‐based agrarian diet, rich in fruit and legume fibre (2–6 years old, 12.6 g/14.2 g total fibre), low in fat and animal protein, was compared to age‐matched children living in European Union (EU) and consuming a ‘Western’ diet rich in animal fat and low in fruit and legume dietary fibre (2–6 years old, 3.3 g/8.6 g total fibre). 16S rRNA sequence analysis revealed significant differences between the two groups, particularly with respect to Actinobacteria, Bacteroidetes and Firmicutes. Faecal microbiota of the Burkina Faso children was rich in Actinobacteria and Bacteroidetes but had lower levels of Firmicutes. Conversely, EU children were rich in Proteobacteria and had over twice the relative abundance of Firmicutes to Bacteroidetes [EU, 2.8 ± 0.06 (F/B ratio ± s.d.) vs. African, 0.47 ± 0.05; *P < *0.001]^33^; see Figure 1. Of note too, the African children exhibited increased richness and biodiversity in their faecal microbiota compared to that identified in European children, with unique abundance of Bacteroidetes genera *Prevotella* and *Xylanibacter* and Spirochaetes of the genus *Treponema* not found in European faecal samples. These bacteria, which possess enzymes relevant to cellulose and xylanhydrolysis, are capable of metabolising plant cell wall dietary fibre and generating significant levels of secondary fermentation products, particularly SCFAs. In support of this, solid phase micro‐extraction gas chromatography mass spectrometry analysis revealed that faecal levels of total SCFA were high in the African children [67.8 ± 12.8 μmol/g faeces (mean ± S.E.M.) vs. EU, 30.14 ± 4.4; *P *≤* *0.001). Specifically, propionic and butyric acid levels were greatly enhanced (~fourfold for both) compared to European faecal samples (e.g. African, 9.25 ± 1.9 μmol/g faeces vs. EU, 2.50 ± 0.5; *P *≤* *0.001). The authors hypothesised that the high colonic SCFA concentrations found, could inhibit the growth of potentially pathogenic Enterobacteriaceae, such as *Shigella* spp. and *Escherichia* spp., which were significantly under‐represented in faecal samples of the African children^33^; see Figure 1. Similar studies comparing agrarian societies relative to those individuals living in Westernised societies, have also reported differing microbiome patterns with agrarian diets producing higher faecal levels of SCFAs^52^, ^53^, ^54^; see Table 2.

**Table 2:** Population studies examining effect of long‐term (habitual) diet on human gut microbiome and metabolome

Study (reference)	Population	Subjects (*n*)	Major dietary component (fibre intake)	Predominant microbiota (relative proportions)	SCFAs (*P* value)	
De Filippo *et al*.^33^	Burkina Faso	15 healthy (1–6 years)	1–2 years: breast milk, cereals, fruit (10.0 g/day fibre); 2–6 years: fruit, legumes (14.2 g/day fibre)	Bacteroidetes (58%) Actinobacteria (10%)	↑ Total (*P < *0.001)	↑ Acetate (*P < *0.01) ↑ Valerate (*P < *0.01) ↑ Propionate (*P < *0.001) ↑ Butyrate (*P < *0.001)
	Italy (EU)	15 healthy (1–6 years)	1–2 years: Breast milk/milk, cereals, vegetables/fruits, meat (5.6 g/day fibre); 2–6 years: Cereals, vegetables, fruits, cow's milk, meat, fish, egg (8.4 g/day fibre)	Firmicutes (64%) Proteobacteria (7%) (incl. *Escherichia* and *Shigella* spp.)	↓ Total (*P < *0.001)	↓ Acetate (*P < *0.01) ↓ Valerate (*P < *0.01) ↓ Propionate (*P < *0.001) ↓ Butyrate (*P < *0.001)
Yatsunenko *et al*.^52^	Malawi	115 healthy (0–70 years)	From breast milk to maize > cassava and other fruit/legume polysaccharides	*Prevotella* spp.	Not studied N/A	
	Venezuela	100 healthy (0–70 years)	Maize, cassava and other plant polysaccharides	*Prevotella* spp.		
	United States	316 healthy (0–70 years)	Western style – no specific reference to diet composition	*Bacteroides* spp.		
Lin *et al*.^53^	Bangladesh	6 healthy (8–13 years) 4 healthy (18–41 years)	Rice, bread and lentils little meat – no specific data	Firmicutes (60%) Bacteroidetes (20%) esp. *Prevotella* spp. Tenericutes (12%) esp. *Bifdo*. spp. Proteobacteria (7%)	Not studied N/A	
	United States	4 healthy (10–14 years)	More diverse diet – high animal fat and protein, carbohydrates, vegetables – no specific data	Firmicutes (46%) Bacteroidetes (43%) esp. *Bacteroides* spp. Tenericutes (4%) Proteobacteria (4%)		
Ou *et al*.^54^	Africa	12 healthy (50–65 years)	Protein: 58 g/day; Fat: 38 g/day Carbohydrate: 282 g/day (17 g/day fibre)	Bacteroidetes: ↑ *Prevotella* spp. (11%) Firmicutes: *F. prausnitzii* spp. (0.7%)	↑ Total (*P < *0.05)	↑ Butyrate (*P < *0.05) ↑ Acetate (*P < *0.05) ↑ Propionate (*P < *0.05)
	United States	12 healthy (50–60 years)	Protein: 94 g/day; Fat: 114 g/day Carbohydrate: 312 g/day (20 g/day fibre)	Bacteroidetes: ↑*Bacteroides* spp. (24%) Proteobacteria: ↑*Escherichia* and *Acinetobacter* spp.	↑ Isobutyrate (*P < *0.02) ↑ 2‐methyl butyric/isovaleric acid (*P < *0.002)	

**Figure 2:**
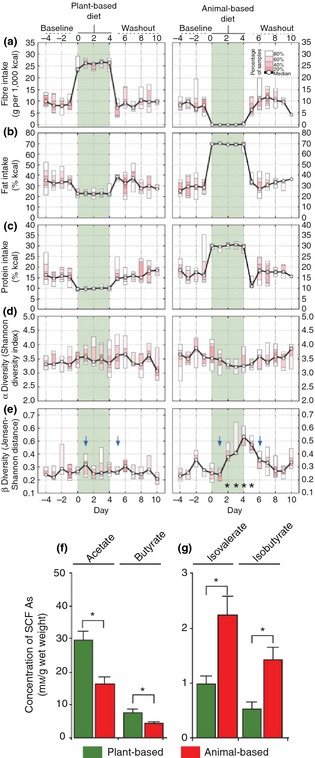
Short‐term dietary intervention alters the human gut microbiota and microbial activity. Ten subjects were tracked across each diet arm. (a) Fibre intake on the plant‐based diet (rich in grains, legumes, fruits and vegetables) increased (*P *=* *0.007; two‐sided Wilcoxon signed‐rank test) but was negligible on the animal‐based diet (meats, eggs and cheeses). (b) Daily fat intake doubled on the animal‐based diet (*P *=* *0.005), but decreased on the plant‐based diet (*P *=* *0.02). (c) Protein intake also rose on the animal‐based diet (*P *=* *0.005), and decreased on the plant‐based diet (*P *=* *0.005). (d) Microbial diversity within each subject at a given time point (α diversity) did not significantly change during either diet. (e) However, the similarity of each individual's gut microbiota to their baseline communities (β diversity) decreased on the animal‐based diet (dates with *q *< 0.05 identified with asterisks; Bonferroni‐corrected, two‐sided Mann–Whitney *U*). Community differences were apparent 1 day after a tracing dye showed the animal‐based diet reached the gut (blue arrows depict appearance of food dyes added to first and last diet day meals). (f) The plant‐based diet generated higher levels of short‐chain fatty acid (SCFAs) typical of plant fibre polysaccharide fermentation than that of the animal‐based diet. (g) Products of dissimilatory amino acid metabolism (branched‐chain SCFAs) by colonic microbiota were seen on the animal‐based diet (*P <* *0.05, two‐sided Mann–Whitney *U*;* n* = 9–11 faecal samples per diet arm).^59^ Reproduced with permission from Macmillan Publishers Ltd: *Nature*, copyright 2014.

In support of the data coming from global population studies, Wu *et al*.^55^ also evaluated the effect of dietary fibre consumption on the intestinal microbiota composition, and reported similar results. Using recent and long‐term dietary questionnaires and 16S rRNA sequencing to characterise faecal samples from 98 healthy human subjects, microbiota taxa analysis demonstrated that diet low in fat and high in dietary fibre was associated with higher Firmicutes, but diet high in fat was more highly associated with Actinobacteria and Bacteroides. There was a greater *Prevotella*:*Bacteroides* ratio with respect to those consuming a high dietary fibre‐rich and/or vegetarian diet typical of agrarian societies. By contrast, diets high in fat and animal protein and low in dietary fibre, similar to a Western diet, showed the opposite association.^55^ There has been growing concern that even short‐term dietary changes, particularly to a ‘Westernised’ style diet (high animal fat, high sugar and low in plant‐based fibre) can rapidly alter the composition and metabolic activity of resident intestinal microbiota micro‐organisms. This has been seen in several animal models,^56^, ^57^, ^58^ with decreased levels of beneficial Firmicutes and increased numbers of bile‐tolerant, inflammation‐associated Proteobacteria (e.g. *Bilophila* spp. and adherent, invasive *E. coli*).^57^, ^58^ In humans also, short‐term consumption of diets that are exclusively animal (protein and fat) or plant‐based have major effects^59^; see Figure 2. An animal‐based diet was seen to increase abundance of bile‐tolerant, inflammation‐associated bacteria, including *Bacteroides* and *Bilophila*, and reduce levels of the Firmicutes needed to metabolise plant fibre.^59^ In concert, lower concentrations of SCFAs (butyrate and acetate) typical of plant fibre polysaccharide fermentation were observed following ingestion of the animal‐based diet with significantly greater emphasis on dissimilatory branched‐chain amino acid metabolism by colonic bacteria; Figure 2.

Diet (and dietary fibre intake) also has major influence on the intestinal microbiota within the ageing gut. Dietary choice and malnutrition, failing health and immobility were associated with loss of microbial diversity. In 178 elderly subjects [mean age of 78 (±8 s.d.)], either community dwelling, attending an out‐patient day hospital, in short‐term (<6 weeks) rehabilitation care or in long‐term residential care, food frequency questionnaire (FFQ) dietary data was collected and correlated with changes in faecal stool microbiota as analysed by 16S rRNA sequencing.^60^ While the composition of the intestinal microbiota in older subjects (>65 years) exhibited extreme inter‐individual variation, significant differences were identified. Of significant note, differences occurred between those living in community residence (98% of which consumed a low‐fat/high‐fibre or moderate‐fat/high‐fibre diet) and those in long‐term residential care (83% of which consumed a moderate‐fat/low‐fibre or high‐fat/low‐fibre diet). Microbiota of community‐dwelling subjects exhibited increased microbiome richness, particularly high proportion of Firmicutes, while those in long‐term care exhibited lower bacterial richness with a higher proportion of Bacteroidetes. The faecal metabolome was also closely related to community setting, with SCFAs butyrate, acetate and propionate at a higher abundance in community‐dwelling subjects. In addition, shotgun metagenomic sequencing revealed significantly higher gene counts and coverage for butyrate‐ and acetate‐producing enzymes in community dwelling in comparison to long‐stay subjects. Importantly, the microbial changes reported in this study also had a significant impact on human health. Markers of inflammation, such as tumour necrosis factor (TNF‐α), interleukins IL‐6 and IL‐8 and C‐reactive protein, were significantly elevated in long‐stay subjects. They also scored poorly for a range of diverse health parameters.^60^

### Intervention studies

The effect of dietary fibre on the intestinal microbiota has also been investigated in controlled dietary intervention studies. While short‐term intervention studies do indicate significant and rapid effect on the composition of the intestinal microbiota,^55^, ^56^, ^61^ the response appears much more modest, less permanent and with higher inter‐subject variability than that of long‐term, habitual diet. Nevertheless, short‐term dietary intervention has shown significant alteration of the intestinal microbiota. These profound effects include those seen with ingestion of depleted carbohydrate diets, which typically involve some reduction in dietary fibre [including fermentable oligo‐, di‐ and monosaccharides and polyols (FODMAPs) restriction or exclusive enteral feeding], as well as enrichment diets, which involve supplementation with dietary fibre nondigestible oligosaccharides, RS or NSPs.

#### Carbohydrate‐restricted diets

Decreased total carbohydrate intake, as seen in weight‐loss diets, is typically accompanied by some reduction in dietary fibre. Provision of such diets to overweight and/or obese volunteers has been shown to alter bacterial populations in the large intestine.^62^, ^63^, ^64^ Short‐term dietary changes tend to produce relatively modest transient changes at best, although severe energy restriction (by 35% for 6 weeks) has been shown to increase bacterial diversity, particularly among those who start from a low level of diversity.^61^

In a large parallel group study performed by Brinkworth *et al*.,^63^ 91 overweight and obese human volunteers were randomly assigned to an 8‐week energy‐restricted (~30%) diet of low‐carbohydrate (4% of total energy), high‐fat (LC) diet or to a high‐carbohydrate (46%), low‐fat (HC) diet. FFQ and faecal stool was taken at baseline (week 0), and another stool sample after intervention (week 8) with SCFAs analysed bacterial composition determined by selective plating. Although total enumerated anaerobe:aerobe was unchanged on either diet, there was a significant fall in Bifidobacteria numbers (−1.7 ± 1.2 log_10_ cfu/g faeces; *P < *0.001) on the LC diet, but Lactobacilli numbers were unchanged. Total faecal content SCFA levels were also seen to be lower on the LC diet at week 8 compared to week 0 [86.4 ± 45.8 mmol/L (mean ± s.d.) vs. 102.2 ± 33; *P* ≤ 0.04] and HC at week 8 (114.5 ± 38.0). Significant reduction in butyrate (−3.9 ± 9.7 mmol/L; *P *=* *0.001) and acetate (−10.7 ± 26.6 mmol/L; *P < *0.04) at week 8 on the LC diet.^63^

Smaller controlled studies have also reported changes in the intestinal microbiota composition, as well as decreased SCFA concentrations, in response to a dietary intake low in carbohydrate. In a key randomised crossover study by Duncan *et al*.,^62^ 19 healthy obese subjects (BMI range 30–42) initially received a control diet low protein (13%), rich in carbohydrate (52%) for 3 days, followed by 4 weeks on a HPMC diet high in protein (30%) with moderate carbohydrate (35%) or a HPLC diet high in protein, low in carbohydrate (4%) again for 4 weeks.^62^ The two 4‐week test diets were crossed over following 3 days maintenance on the control diet. Enumeration of bacteria in faeces using specific 16S rRNA‐targeted fluorescence *in situ* hybridisation (FISH) probes identified that total bacteria numbers were greater with the control diet vs. the other two diets (control 10.71 log_10_ cfu/g faeces vs. 10.55 and 10.56 for HMPC and HPLC respectively; *P < *0.001). With a reduction in dietary carbohydrate intake, there was also a corresponding decrease in the abundance of *Roseburia* spp. and *Eubacterium rectale* (with control diet rich in carbohydrate at 11.4% of total bacteria; HPMC, 7.8% and HPLC, 3.3%; *P < *0.001) and in *Bifidobacterium* spp. (control 4.0%; HPMC, 2.1% and HPLC, 1.9%; *P < *0.05). Total SCFA were also reduced in response to lowering carbohydrate intake (control, 114 mmol/L; HPMC, 74 mmol/L and HPLC, 56 mmol/L; *P < *0.001) with a disproportionate decrease in faecal butyrate (18 and 9 mmol/L respectively; *P < *0.001) as other major SCFAs acetate and propionate were increased or unaltered respectively.^62^ Bacteria closely related to *Roseburia* spp. and *E. rectale* have been shown to hydrolyse carbohydrates such as starch, xylan and inulin for their growth^62^ and *in vitro* studies have suggested that butyrate is the predominant fermentation product.^62^, ^65^ This could explain the correlation between the change in microbiota and the corresponding decrease in the levels of SCFA, particularly butyrate, following the consumption of a low‐carbohydrate diet. A similar, but smaller randomised crossover study further examined reduced carbohydrate weight‐loss diets for their effects on microbiota‐derived metabolites relevant to colonic health.^64^ Here, 17 obese males (BMI range 30–48.5) were provided control diet for 7 days, followed by 4 weeks on a HPLC or HPMC diet as per Duncan *et al*.^62^ With lowering carbohydrate intake, there was a dose‐dependent decrease in abundance of *Roseburia* spp. and *E. rectale* (*P *<* *0.001) as well as 22% reduction in *Bacteroides* spp. numbers (*P *<* *0.01). Faecal SCFA concentrations were significantly lower on the HPLC diet (*P < *0.001), particularly levels of butyrate (control diet, 17 mmol/L; HPMC, 15 mmol/L and HPLC, 9 mmol/L; *P < *0.001). Ingestion of the HPLC diet significantly decreased levels of plant cell wall‐derived phenolic compounds with known anti‐inflammatory properties, e.g. ferulic acid (<3% of levels seen on control diet; *P *<* *0.001), and increased levels of potentially hazardous faecal water metabolites, including phenylacetic acid (HPMC 63 mmol/L and HPLC 44 mmol/L vs. control, 23.5 mmol/L; *P < *0.001) and total *N*‐nitroso compounds (1474 and 2203 vs. control, 405 ng/mL; *P < *0.001). The pro‐carcinogenic properties of these metabolites found increased in the faecal water following carbohydrate restriction also suggests that long‐term adherence to this style of diet could have a negative impact for maintenance of colonic health.^64^

FODMAP restriction diets are also increasingly being applied as first‐line therapy for gastrointestinal symptom relief, particularly for patients with irritable bowel syndrome (IBS). These diets also have marked effects on intestinal microbiota composition.^66^, ^67^ Short‐chain FODMAPs are also substrates for fermentation by bacteria but have not been generally been considered to be ‘prebiotic’ as per Bifidogenic fructo‐oligosaccharides (FOS) and galacto‐oligosaccharides (GOS); as discussed later. In 2012, a randomised parallel group study reporting IBS symptom relief in 19 patients ingesting a dietitian‐taught fermentable carbohydrate restriction diet [mean total fermentable carbohydrate 17.75 (95% CI, 14.4–21.7) g/day] for 4 weeks compared to 22 individuals on a habitual UK diet [29.65 (24.5–35.7) g/day] (*P *=* *0.005) showed significant reduction in the proportion (and levels) of faecal *Bifidobacterium* spp. [% of total bacteria, 3.2 (1.8–5.8) vs. 0.54 (0.2–0.9) respectively; *P *<* *0.001].^66^ This reduction in Bifidobacteria may have been a consequence of ~50% lower daily intake of both prebiotic FOS and GOS in the intervention group. Total microbiota levels, *Lactobacillus*–*Enterococcus, Bacteroides*–*Prevotella*,* E. rectale*–*C. coccoides* and *Faecalibacterium prausnitzii*, were all similar at baseline (in both groups) and all unchanged following intervention. This likely reflected no overall changes observed in faecal pH or total SCFA levels.^66^

A recent randomised controlled efficacy trial by Halmos *et al*.^67^ of two diets over 3 weeks, a low‐FODMAP diet [total FODMAP intake 3.05 (1.86–4.25) g/day] compared to a typical Australian diet higher in FODMAP content [23.7 (16.9–30.6) g/day], included patients with IBS and healthy subjects of similar demographics and habitual diet intake.^67^ After 21 days on the diet, each participant undertook a ‘washout’ period of at least 21 days in which they then resumed their usual habitual diet and then crossed over to the alternate diet. Here, *Bifidobacterium* spp. were similar between the two diets despite greater diversity of butyrate‐producing microbiota clusters, and reduced overall bacterial abundance [9.63 (9.53–9.73) log_10_ 16S rRNA gene copies/g faeces; *P *<* *0.001] on the low‐FODMAP diet compared to that seen on the high‐FODMAP Australian diet [9.83 (9.72–9.93)]. In relation to participant habitual diet, the low‐FODMAP diet reduced total bacterial abundance, while the higher FODMAP‐containing typical Australian diet increased relative abundance for butyrate‐producing bacteria, e.g. Clostridium cluster *XIVa* (median ratio 6.62; *P *<* *0.001) and mucus‐associated, mucin oligosaccharide degrading *Akkermansia muciniphila* (19.3; *P *<* *0.001). Again, no alterations in faecal SCFA levels were observed with the different FODMAP diets ingested over 3 weeks although somewhat surprisingly, the lower FODMAP diet was associated with higher faecal pH (7.37 vs. 7.16 for the typical Australian diet and 7.18 following a habitual diet; both *P *=* *0.01). It is clear that additional short‐term and long‐term interventions studies are needed to assess the functional significance and health implications of such intervention in treating patients, and their use in asymptomatic healthy populations.

Exclusive enteral nutrition can also be effective as primary therapy in children and adolescents with Crohn's disease^68^ and although used less frequently in adult Crohn's, when assessed using high‐quality studies only, the results are similar to those achieved with corticosteroids.^69^ There is little understanding of how it works but one plausible mechanism could simply be through starving the intestinal microbiota of nutrients, perhaps particularly distal ileal bacteria. In an intriguing study by D'Argenio *et al*.,^70^ examining the ileal mucosa‐associated microbiota in a teenager with Crohn's disease following enteral nutrition as sole therapy, it was shown that induction of remission was accompanied by normalisation of the ileal microbiota.^70^ Conversely, but not necessarily in contradiction to this, enteral nutrition is associated with a reduction in faecal microbiota diversity and reduction in potentially beneficial *F. prausnitzii*.^71^ Likewise, further studies are clearly needed to understand the mechanisms underlying effectiveness of a specific carbohydrate exclusion diet (restricting intake of complex carbohydrates and eliminating refined sugar) that resulted in clinical and mucosal improvement of children with Crohn's disease maintained on this diet for 12 weeks, with sustained improvements seen for those continuing on the diet for 52 weeks.^72^ Restricting intake of complex carbohydrates, known to be fermentable by Firmicutes to mucosa‐beneficial SCFAs, would perhaps seem counterintuitive, but the elimination of refined sugar may perhaps be more important, reducing mucosal association of pro‐inflammatory Proteobacteria known to be increased in numbers in the mucosae of adult^73^, ^74^ and paediatric Crohn's patients,^75^, ^76^ and in mice fed a Westernised diet, high in fat and rich in refined sugar.^58^

#### Enrichment with prebiotics

While a low overall carbohydrate intake in the diet causes changes in the gut microbiota that could potentially have a negative impact on health, dietary intervention studies indicate that supplementation with dietary fibre can alter the microbiota in a more beneficial fashion. Prebiotics are nondigestible dietary fibres that confer benefit to host intestinal health by selectively stimulating growth of a limited number of indigenous bacteria, particularly but not exclusively, *Bifidobacterium* and/or *Lactobacillus* spp.^42^, ^77^, ^78^ Such benefits include enhancement in gut mucosal barrier integrity and function, increased host mucosal immunity, increased SCFA production and an associated reduction in mucosal interaction of opportunistic enteric pathogens.^78^, ^79^

The prebiotic effect of dietary oligosaccharides inulin and oligofructose has been extensively studied *in vivo*. Early studies were typically performed with supplemented diets in germ‐free rodent models inoculated with faecal microbiota from human donors, which then develop an established microbiome similar to that of a mature human adult.^80^, ^81^, ^82^, ^83^ In the study by Kleessen *et al*.,^82^ where human flora‐associated (HFA) rats were provided a standard chow diet supplemented with either 50 g/kg short‐chain oligofructose, long‐chain inulin or a 50:50 mix over 7 days (consumption ~23–24 g/day), a Bifidogenic effect was observed in the colon of those on the diet containing oligofructose alone (*P < *0.005) and in the caecum of animals on the mixed diet. Those animals also exhibited higher caeco‐colonic numbers of Lactobacilli (*P < *0.05) in comparison to HFA rats fed standard diet, as well as significantly smaller numbers of caecal, colonic and faecal bacteria belonging to potentially pathogenic *Clostridium hystolyticum* and *C. lituseburense* groups (6.8 and 6.9 vs. 7.9 log_10_ counts/g wet weight). While each diet had a variable Bifidogenic effect, their effect on generation of caeco‐colonic pH and SCFA generation was more consistent. Caecal and colonic pH was lower (*P *<* *0.05, excepting the mix diet) and levels of butyrate were increased in response to each of the three diets (all *P *<* *0.05). Faecal levels of butyrate were also elevated by all three test diets but only significantly in HFA rats consuming the two inulin‐containing diets (*P < *0.05).^82^

**Figure 3:**
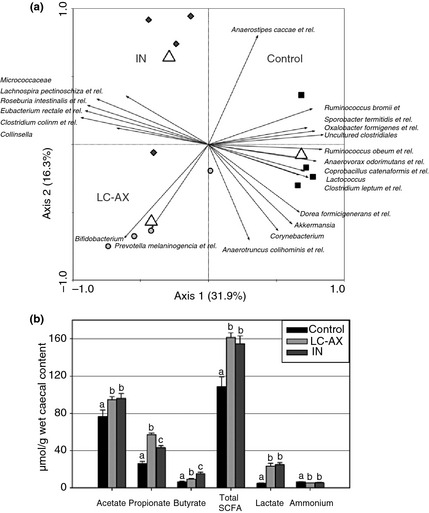
Prebiotic intervention with arabinoxylans and inulin differentially modulate the mucosal and luminal gut microbiome and metabolome of humanised rats. (a) The intestinal microbiota at the site of fermentation (caecum) in humanised rats fed a diet supplemented with long‐chain arabinoxylan (LC‐AX) or inulin (IN) is significantly different to those fed control diet (*P *=* *0.002). The redundancy analysis (RDA) at bacterial group‐level is based on the human intestinal tract (HIT) Chip microarray data performed on samples from final day of intervention (*n *=* *4). Of 131 bacterial groups identified with HITC hip, 22 groups were retained (average cumulative abundance of these 22 groups = 42%) explaining 31.9% of the variation between these diets along the *x*‐axis and 16.3% of the variation along the *y*‐axis. (b) Absolute levels of SCFA (total and individual; μmol/g wet caecal content) at the end of the intervention were increased in the caecum of humanised rats fed LC‐AX or IN (*n *=* *8), whereas ammonium levels (indicative of protein metabolism) decreased. Values indicated with a different superscript are significantly different (a, b or c).^83^ Reproduced from Van den Abbeele *et al*. *Environmental Microbiology* 2011; 13(10): 2667–80. with permission of Wiley.com.

In a more recent study,^83^ where HFA rats were given diet containing 10% w/w inulin or prebiotic arabinoxylan (replacing 5% each of sucrose and maize in the control diet), consuming an average ~15 g/day for 3 weeks, no increase in *Bifidobacterium* spp. numbers was seen, but there was significant increase in numbers of other key SCFA‐producing bacterial species, including *Roseburia intestinalis* and *E. rectale* (*P < *0.05), with arabinoxylan effecting a 60‐fold increase in Bifidobacteria (*P *<* *0.05). Both inulin and arabinoxylan consumption significantly increased caecal total SCFA concentrations (*P < *0.05), with caecal pH also significantly decreased from control^83^; see Figure 3. The lack of any Bifidogenic effect with inulin in this study is in contrast to an earlier study.^84^ It has recently been proposed that the variable effects observed between studies may be due to differences in inulin structure, as its degree of polymerisation (2–60 units of β2‐1 linked fructose) and thus molecular weight can vary significantly according to choice of plant source, growing and harvesting conditions.^85^ It has also been suggested that Bifidobacteria may not be able to efficiently degrade long‐chain inulins due to a lack of appropriate enzymes.^78^ Furthermore, it is likely that the numbers of *Bifidobacterium* spp. initially present in the intestine of rats with a huma nised microbiota could influence strongly the magnitude of any prebiotic‐driven Bifidogenic response.^82^

With the advent of high‐throughput sequencing techniques, a number of key human intervention studies using prebiotics have now been conducted (discussed below) to examine effects on the intestinal microbiota, from a community‐wide and species‐specific perspective. Prebiotic supplements typically exhibit a substantial Bifidogenesis, even at levels of consumption as low as 4 g daily.^86^

Prebiotic lactulose (a synthetic disaccharide, which humans are incapable of digesting) has also been shown to effect Bifidogenesis and growth of lactic acid bacteria in the colon when supplemented to diets.^87^ In a human volunteer study,^88^ 20 subjects randomly assigned to two equal‐sized groups were given either a lactulose powder supplement (10 g/day) or placebo (5 g glucose, 5 g lactose/day) for 26–33 days. Faecal stool sampling was performed before, towards the end of treatment (last 2–3 days) and 26–33 days post‐treatment. While no differences in total bacteria were seen on either supplement, bacterial enumeration by FISH and culture methods showed that lactulose‐treated volunteers had increased levels of *Bifidobacterium* spp.(9.3 ± 0.3 log_10_ bacteria/g faeces; mean ± s.d.) above that observed in pre‐treatment (8.8 ± 0.5; *P *<* *0.01) and placebo control samples (data not provided; *P *<* *0.01). Post‐lactulose treatment (at 60 days), levels of Bifidobacteria fell back to pre‐treatment levels.^88^

The prebiotic action of inulin was also examined in a recent double‐blind, placebo‐controlled, crossover study^89^ with 32 healthy adults (20–42 years) allocated to two groups consumed 10 g/day of either very long‐chain inulin (VLCI; extracted from globe artichoke *Cynarascolymus*) or maltodextrin placebo for 3 weeks, followed by 3‐week wash out and then 3 weeks on the alternative test diet. FISH analysis of bacteria group abundance in faeces indicated that total bacteria numbers were unaffected, but that consumption of VLCI resulted in Bifidogenesis (2.82‐fold increase before intervention and 2.75‐fold increase following placebo; both *P *<* *0.05). Lactobacilli also increased (2.42‐fold pre‐inulin; 5.88‐fold post‐maltodextrin; both *P *<* *0.05), while *Bacteroides*–*Prevotella* numbers were significantly reduced (1.77‐fold decrease; *P < *0.05) in comparison to placebo. In contrast, there were no significant changes in the concentration of faecal SCFA, but there were increased symptoms of bloating recorded on the VLCI diet.^89^

Similar results have been observed in a double‐blind, randomised, placebo‐controlled parallel group study each with 15 healthy volunteers ingesting vegetable snack bars with 7.7 g/day inulin derived from either Jerusalem artichoke (*Helianthus tuberosus*), chicory (*Cichoriumintubus*) or a cereal mixture (placebo).^90^ Subjects consuming inulin from either source, showed a gradual Bifidogenic effect over the 3‐week intervention period, with significant differences observed in faeces at end of week 1, 2 and 3 compared to placebo (+1.2 log_10_ cfu/g faeces at 3 weeks, *P < *0.05). In addition, there was significant reduction in *Bacteroides*–*Prevotella*, and a lower frequency of *Clostridium histolyticum/C. lituseburense* (both *P < *0.05). Here again, no significant changes were observed in SCFA levels following inulin supplementation.^90^

Selective enrichment of particular *Bifidobacterium* spp. by prebiotics has also been investigated. Ramirez‐Farias *et al*.,^91^ using a quantitative real‐time PCR approach, investigated which particular species of *Bifidobacterium* were increased in response to inulin supplementation. In their balanced crossover study, 12 healthy adults were randomly split into two groups either consuming inulin (5 g twice daily) over 21 days or those that received no supplement. Daily consumption of inulin (faeces sampled at day 16) significantly increased relative abundance of faecal *Bifidobacterium* spp. in comparison to the no supplement controls (*P < *0.001). Specifically, qPCR analysis showed selective enrichment of a number of distinct lineages, namely *Bifidobacterium adolescentis* (>fourfold increase, *P < *0.001), *B. bifidum* (2.7‐fold, *P < *0.001) and *B. longum* (*P *=* *0.055). However, the level inter‐individual variation, both baseline abundance of Bifidobacteria and the magnitude of response to the inulin supplement, was observed to be high. Levels of the beneficial, butyrate‐producing Firmicute *F. prausnitzii* was also significantly elevated in all subjects consuming inulin (14.5% of total bacteria vs. 10.3% for controls; *P < *0.05), but response was dependent on order of inulin intervention.^91^

Similarly, in a later study by Davis *et al*.,^92^ where 18 healthy human subjects were given caramel chews containing prebiotic GOS (0–10 g/day) each for 3 weeks, weekly faecal analysis by pyrosequencing of 16S rDNA tags showed that consumption of >5 g/day GOS increased abundance of Bifidobacteriaceace (*P *<* *0.0001). Several distinct lineages of *Bifidobacterium* were observed to be enriched, notably *B. adolescentis*,* B. longum* as well as *B. catenulatum* (each three‐ to fourfold increase; *P *<* *0.05). This was paralleled by significant decreases observed within the Bacteroidaceae family (*P < *0.01), in particular within the genus *Bacteroides* (22% decrease; *P *<* *0.0001) both when compared to the non‐GOS controls. It must also be noted that considerable subject to subject variation was seen with the GOS supplement intervention, particularly at higher doses. Some individuals were noted to be unaffected by GOS consumption.^92^ Likewise, a recent clinical trial by Joossens *et al*.,^93^ examining 17 healthy individuals who received oligofructose‐enriched inulin (20 g/day for 4 weeks) also showed species‐specific increases in *B. longum* (*P *<* *0.003) and *B. adolescentis* (*P *<* *0.02) using denaturing gradient gel electrophoresis (DGGE) to study faecal microbiota diversity, with differences confirmed by qPCR (both *P *<* *0.05).^93^

With selective enrichment of *Bifidobacterium* spp., there is often observed decrease in *Bacteroides* numbers.^89^, ^90^, ^91^, ^92^ Likely, selective enrichment of Bifidobacteria and other key butyrogenic species, including *F. prausnitzii*,^83^, ^91^, ^94^ in response to prebiotic ingestion leads to a fall in colonic pH, thereby inhibiting the growth of pH sensitive species within key bacterial phyla (including *Bacteroides* spp. and opportunistic Gram‐negative pathogens.^46^, ^95^ However, over 95% of SCFAs produced in the human large intestine are thought to be rapidly absorbed, meaning that only a small proportion of SCFAs are likely excreted in the faeces.^89^, ^90^ Human faeces samples might not accurately reflect SCFA production in the colon, and it is perhaps unsurprising that none of the studies described here have reported any change in faecal SCFA concentration.

While studies have shown that supplementation with prebiotics can stimulate the relative abundance of *Bifidobacterium* spp. and potentially contribute to the suppression of potential pathogenic bacteria, it must also be noted that considerable inter‐individual variation has been observed in these studies, with some volunteers identified as ‘nonresponders’.^91^, ^92^ These results highlight that prebiotic responses are not universal, and they are also influenced by the initial composition of an individual's microbiota.^96^ In addition, all these studies described were based on conventional 16S rRNA gene‐based microbial profiling, and *Bifidobacterium* spp. are known to be poorly differentiated, and levels therefore significantly underestimated, without application of more precise targeted amplicon approaches as recently described.^97^ Therefore, studies involving larger numbers of volunteers, together with more detailed analysis of the microbiota might be required to fully elucidate and understand the action of dietary prebiotics on the global gut microbiome.

Despite these drawbacks, it is certainly clear that dietary oligosaccharides have the potential to selectively alter the gut microbiota composition, and could therefore act as a therapeutic agent to treat dysbiosis in the intestinal microbiota. Indeed, a number of recent human clinical feeding trials have been performed to assess the efficacy of prebiotics in the treatment of, and correction of the dysbiosis seen in, IBD^98^, ^99^, ^100^, ^101^, ^102^, ^103^, ^104^; see Table 3.

**Table 3:** Results of clinical trials of prebiotics in IBD

Patients (*n*) (disease activity)	Intervention (dose/duration)	Trial type	Primary endpoint	Results (*P* value)	Microbiota changes	Metabolome changes (faecal pH/SCFAs)	Reference
UC (29) (remission)	Ispaghula husk (lactose‐free) (8 g/day; 6 months)	Open‐label	Rate of relief of GI symptoms	69% improved with active, 24% placebo (*P *<* *0.001)	n.d.	n.d.	Hallert *et al*.^98^
UC (21) (mild/moderate)	Germinated barley (20–30 g/day; 24 weeks)	Open‐label	CAI	Reduced clinical activity over 24 weeks (*P *<* *0.05)	n.d.	n.d.	Kanauchi *et al*.^99^
UC (59) (remission)	Germinated barley (20 g/day; 12 months)	Open‐label	CAI and endoscopic index	Better maintenance of remission up to 12 mo (*P *<* *0.05)	n.d.	n.d.	Hanai *et al*.^100^
IBD (14 UC, 17 CD) (‘mostly active’)	Lactulose (10 g/day; 4 months)	Open‐label	CAI and endoscopic score	No improvement in UC or CD activity scores, some improvement in QOL in UC (n.s. excepting QOL) (*P *=* *0.04)	n.d.	Faecal pH (↑ UC n.s.) (↔ CD)	Hafer *et al*.^101^
CD (103) (active)	Fructo‐oligosaccharides (FOS) (15 g/day; 4 weeks)	Double‐blind	70 point fall in CDAI	FOS 22% response, placebo 39% response (*P *=* *0.67 favouring placebo)	↔*Bifido*. spp. (*P *=* *0.201) ↔*F. prau* (*P *=* *0.95)	n.d.	Benjamin *et al*.^102^
CD (67) (inactive and moderately active)	Oligofructose‐enriched inulin (20 g/day; 4 weeks)	Double‐blind	Metabolite profiles	Clinical secondary outcomes: median HBI reduced from 4 to 3 active vs. 4 to 4 in placebo (*P *=* *0.048)	↑*Bifido. longum* (*P *=* *0.03) ↓*Rumino. gnavus* (*P *=* *0.03)	↑ butryate (*P *=* *0.0011)↑ acetaldehyde (*P *=* *0.0008) also indicative of carbohydrate fermentation	Joossens *et al*.^103^ De Preter *et al*.^104^

IBD, inflammatory bowel disease; CD, Crohn's disease; UC, ulcerative colitis; CAI, Clinical Activity Index; CDAI, Crohn's Disease Activity Index; HBI, Harvey Bradshaw Index; n.d., no data; n.s., nonsignificant change; QOL, quality of life.

#### Enrichment with RS

Resistant starch, a valuable component of dietary fibre intake, is defined as starch that escapes digestion in the small intestine and provides a source of fermentable substrate for caecal and colonic microflora.^105^ RS can be classified into four subtypes (RS1–4): RS1, physically inaccessible starch granules locked within whole grains (or partially milled grains) and legumes: RS2, granular starch that is tightly packed, consisting of ungelatinised granules, as in raw potato, tubers, cereals and unripe banana: RS3, highly RS fraction, and is mainly composed of retrograded amylose (formed when cooked and cooled): RS4, starch chemically or enzymatically modified to resist digestion.^106^ The potential of RS, as a key component of dietary fibre and source of SCFAs, to impact on stability and diversity of the intestinal microbiota has mainly been conducted in animal studies with a limited number of human trials. While the results from some of the key studies (detailed below) provide convincing evidence to suggest that RS can modulate the intestinal microbiota, it is worth noting that the specific effect of RS seems to be highly variable between species and individuals.

A number of *in vivo* animal studies have demonstrated that RS has significant prebiotic effect.^81^, ^107^, ^108^, ^109^ In the seminal study conducted by Kleessen *et al*.,^108^ the long‐term effect of diet supplemented with RS1 (from native granular potato starch) or RS2 (modified potato starch) on the intestinal microflora of rats was evaluated over a period of 5 months. Both RS1‐ and RS2‐fed animals demonstrated increased abundance of anaerobes; RS1 and RS2 led to increased *Bifidobacterium* spp. (as assessed by culture, at 5 months), while RS2 consumption alone was seen to enhance colony counts of Lactobacilli, Streptococci and Enterobacteriaceae in the caecum (*P < *0.05). Levels of total caecal SCFAs were increased in RS1 and RS2 fed animals vs. non‐RS controls at 5 months (RS1, 432.2 μmol/g dry weight; RS2, 768.4 vs. 255.2; *P *<* *0.05). Similar responses were seen for faecal SCFAs, with acetate and propionate levels particularly higher.^108^ Other key studies have also demonstrated that RS2 and RS3 consumption raise levels of Bifidobacteria and Lactobacilli, increasing production of SCFAs in the colon, particularly increased propionate and butyrate. Intervention with such RS‐rich diets have been shown to have a protective effect, attributed to observed increased SCFA concentrations, significantly lowering the level of colonocyte DNA damage when compared to higher levels seen for animals fed on a Western style (moderate fat and protein, low RS).^107^

Enhanced butyrate production through long‐term ingestion of RS was elegantly demonstrated in the study conducted by Le Blay *et al*.^110^ Resistant potato starch (RS2) supplements were given to rats, at 90 g/kg for up to 6 months, resulting in elevated levels of butyrate in the caecum and proximal colon (sixfold increase after 6 months compared with 0.5 months; both *P *<* *0.0001) and in the distal colon (up threefold; *P *<* *0.0001).^110^ Other key studies in pigs also confirm that experimental diets containing various sources of RS can increase caecal and colon SCFA levels.^111^, ^112^ Increases in total SCFA are seen in the caeco‐colon within 7 h following ingestion of experimental meals containing either 15 g potato starch (‘RS2’), high amylose maize starch (HS) or retrograded extruded HS (both ‘RS3’), to 33, 78 and 105 mmol/L respectively, with potato starch providing the highest production of butyrate.^111^ In another recent study, 20 female pigs were assigned to a diet high in pre‐gelatinised digestible potato starch (DS) or high in retrograde tapioca starch (RS3) fed over 2 weeks.^112^ RS3 consumption significantly increased both caecal and colonic SCFA concentrations, with the most abundant colonic SCFAs being acetate, propionate and butyrate (*P *≤* *0.05 for all). While RS3 consumption had little effect on relative abundance of both Bifidobacteria and Lactobacilli, instead there was significant increase in *F. prausnitzii* (*P *=* *0.02), with a concomitant decrease in potentially pathogenic members of gamma‐Proteobacteria, including *E. coli* and *Pseudomonas* spp. (*P *=* *0.04).^113^

*In vivo* human studies have also analysed the influence of RS on enhancement of butyrate‐producing microbiota and SCFA production in the intestine.^26^, ^113^, ^114^, ^115^, ^116^ In a small feeding trial, SCFA levels were assessed in 24 healthy subjects consuming each of four supplements (a low‐fibre control diet, and supplements of 30 g wheat bran fibre, RS2 or RS3) for 2 weeks in random order. Both RS diets increased faecal bulking above low‐fibre control albeit less than the wheat bran supplement, and mean faecal butyrate:SCFA ratio was significantly increased by RS diets above that of control by 31 ± 14% (*P *=* *0.035). Using DGGE of 16S rRNA gene fragments, Abell *et al*.^113^ examined for changes in bacterial populations of 46 healthy volunteers in a randomised crossover trial examining intervention diets, one high in RS2 and low NSP (25 g total fibre, 22 g RS) and another high in NSP (25 g total fibre, 1 g RS). The study demonstrated that the faecal microbial community of those consuming RS2 diet was enriched with *Rumincoccus bromii* (67% increase; *P < *0.05), *F. prausnitzii* and *E. rectale*, while consumption of NSP had no effect. However, there was a high level of inter‐subject variation in the bacterial populations examined. Despite this, the presence of *F. prausnitzii* and *E. rectale* was successfully correlated with increased production of all major SCFAs, particularly butyrate, which increased by RS2 rich diet by over 22% (*P *<* *0.001).^113^

Similar results have been demonstrated in other studies, such as that by Walker *et al*.,^26^ who examined the influence of a diet high in RS3 or NSP on the microbiota of 14 overweight males. Stool samples were analysed by 16S rRNA sequencing, and although samples tended to cluster more strongly by individual rather than by diet, ‘blooms’ in specific bacterial groups occurred rapidly, typically being detected within 3–4 days, and reversing equally as fast after the dietary intervention finished. It was observed that relatives of *R. bromii* increased in most volunteers on the RS diet, accounting for an average of 17% of total bacteria, compared with only 3.8% on the NSP diet. In addition, there was also an increase in the abundance of bacteria related to *E. rectale* (10.1% of total bacteria) following RS consumption.^26^

In 2010, Martinez *et al*.^115^ also studied the effect of RS2 and RS4 on the composition of the human microbiota in a placebo‐controlled, double‐blind crossover trial with 10 healthy individuals. RS4, but not RS2, induced phylum‐level changes, significantly increasing Actinobacteria (mean +5%; *P < *0.05) and Bacteroidetes (+5%, *P < *0.01), while decreasing Firmicutes (−10%; *P < *0.001). At the species level, RS4 evoked increases in *B. adolescentis* (10‐fold increase; *P < *0.05) and *Parabacteroides distasonis* (sevenfold increase; *P < *0.001), while RS2 significantly raised proportions of *R. bromii* and *E. rectale* (both *P < *0.05). These substantial shifts in bacterial composition imply that specific bacterial populations have the potential to be selectively targeted by different RS subtypes; however, the study did also report a high level of inter‐subject variation in both the effect of RS, and its magnitude of response. This could be due to the fact that the compositional shifts that occur in the microbiota following RS consumption will depend on its baseline bacterial composition, which tends to vary between individuals.^116^

While *in vivo* human studies have reported a high level of inter‐individual variation, their findings strongly suggest that *R. bromii* relatives could play a key role in RS digestion in the human colon. Further evidence to support this includes *in vitro* fermentation studies, which have shown that *R. bromii* is one of the most predominant bacterial groups to colonise RS.^117^, ^118^ Studies have also shown that bacteria such as *F. prausnitzii* and *E. rectale* commonly increase in dominance following RS consumption. These butyrogenic species are known to substantially contribute to SCFA production, and therefore, together with *R. bromii*‐related phylotypes likely impact to facilitate health‐promoting effects in the large bowel.

#### Nonstarch polysaccharide contrabiotics

*In vivo* human studies provide convincing evidence to suggest that dietary fibre components such as prebiotics and RS can selectively promote growth of specific beneficial bacterial populations, thereby improving intestinal health. In this context, NSPs from a range of sources have been shown to possess limited prebiotic activity.^26^, ^113^ However, studies do suggest that soluble NSPs may interact with the intestinal microbiota in a different manner, via a contrabiotic effect, whereby they prevent potentially harmful interactions between bacteria and the gut epithelium that occur upon dysbiosis.

**Figure 4:**
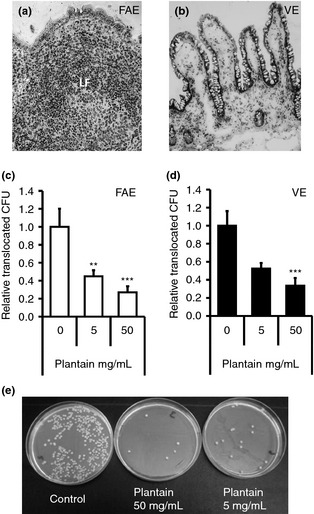
Contrabiotic plantain (banana) NSP blocks translocation of Crohn's disease mucosa‐associated *Escherichia coli* across the human intestinal epithelium. Histology of (a) human villus epithelium (VE) and of (b) an ileal lymphoid follicle (LF) and overlying follicle‐associated epithelium (FAE) following Ussing chamber experiments (×20 magnification). (c, d) Colonic Crohn's *E. coli* isolate HM615 translocation across ileal FAE (*N* = 7) and VE (*N* = 9) is inhibited by 20 min pre‐treatment with plantain NSP. **P <* *0.01; ***P <* *0.001; anova. (e) Overnight culture of Ussing chamber serosal medium following 2‐h translocation of Crohn's disease *E. coli* HM 615 across isolated human epithelium, in the presence and absence of plantain NSP. Reproduced from Roberts *et al*. Gut 2010; 59(10):1331–9, with permission from BMJ Publishing Group Ltd.^119^

In our own studies, a range of soluble plant fibres have been evaluated for their ability to block attachment of adherent, invasive *E. coli* (AIEC), observed in increased number within the mucosae of Crohn's disease and colon cancer patients, to intestinal epithelial cells *in vitro*.^74^, ^119^ Particular efficacy was shown for soluble NSP extracted from plantain bananas (*Musa* spp.), which inhibited AIEC adhesion to, and invasion of, intestinal epithelial cells *in vitro*.^119^ The portal of entry for AIEC is likely through microfold(M)‐cells overlying Peyer's patches in the human ileum and lymphoid follicles in the colon. Importantly, it was demonstrated that AIEC was significantly inhibited across M‐cells modelled *in vitro* as well as across isolated human ileal Peyer's patches mounted in Ussing chambers^119^; see Figure 4. Furthermore, it was shown that soluble NSP from some other sources such as soluble broccoli NSP, but not apple or leek NSP, could also block AIEC–epithelial interaction.^119^

Interestingly, it seems that soluble plantain NSP (particularly the pectic, homo‐galacturonan‐rich polysaccharide components) can also inhibit the adherence of a range of different enteric gut pathogens including *Salmonella* spp., *Shigella* spp., Enterotoxigenic *E. coli* and *C. difficile*.^120^, ^121^ Theses studies also showed plantain NSP blockade of translocation of *Salmonella* Typhimurium across isolated human ileal FAE^120^ and that dietary supplementation of pellet feed with 50 mg/day soluble plantain fibre to feed blocked Salmonellosis in the chicken.^121^ We have suggested that the inhibitory effect of the contrabiotic fibre is mediated by an interaction with the epithelial cell that results in electrogenic chloride secretion, thereby preventing the adhesion of gut pathogens.^121^

Modelling of soluble plantain NSP breakdown using mixed faecal microbiota obtained from healthy volunteers has shown that 25–75% of ingested plantain NSP is likely to avoid fermentation in the human colon.^119^, ^122^ Assuming passage of 1 L of fluid daily into the caecum, we estimated that readily achievable oral dosing of humans with 5 g soluble plantain NSP twice daily would achieve effective luminal concentrations of ~10 and 7.5 mg/mL in the caecum and rectum respectively.^119^ *Escherichia coli*,* C. difficile* and *Salmonellae* certainly interact with soluble plantain NSP and use this as an energy source.^119^, ^120^ However, while *Bacteroides* are also major fermenters of plantain fibre, species from other key genera known to metabolise carbohydrate and plant‐derived polysaccharides, such as *Bifidobacterium*,* Lactobaccillus*,* Streptococcus* and *Ruminococcus,* cannot easily ferment this fibre source^122^ suggesting little or no prebiotic effect for soluble plantain NSP.

While contrabiotics have not yet formally been studied in humans, studies have demonstrated an inverse association between intake of fruit and vegetable fibre and the risk of IBD.^123^ In an analysis of the prospective Nurses’ Health Study, a high‐dietary fibre intake long‐term (170 776 subjects followed up over 26 years, with a FFQ undertaken every 4 years) was associated with a reduced incidence of Crohn's disease, but not ulcerative colitis.^124^ Intake of dietary fibre in the highest quintile, 24.3 g/day, conferred a 40% reduction in risk [multivariate hazard ratio (HR) 0.59, 95% CI 0.39–0.90; *P *=* *0.08] compared to those in the lowest quintile of fibre intake, at 12.7 g/day. Moreover, it was also specifically fibre intake from fruit that had the protective effect, decreasing risk of diagnosis with Crohn's disease by up to half (HR 0.51, 95% CI 0.35–0.76; *P *=* *0.003). No such significant associations in this study were seen for fibre from vegetables [neither total vegetable consumption, nor specifically intake of cruciferous vegetables (i.e. of the genus *Brassica*, including Brussels sprouts, cabbage, kale etc. known to be rich in both soluble fibre and phytochemicals with anti‐cancer properties^125^), nor cereals (whole grain, bran) and legumes]. We suggest that soluble dietary fibres, such as plantain, acting as ‘contrabiotics’ should be studied as a potential prophylaxis or treatment for IBD.

## Conclusions

Long‐term intake of a diet that is high in fruit and legume fibre, typical of those brought up in a rural agrarian community, is associated with greater diversity and marked differences in the faecal microbiota. Identified in a number of recent studies, a high predominance of *Prevotella* to *Bacteroides* is seen in contrast to faecal microbiota of those living in Westernised societies. A ‘Western’, high‐animal fat/high‐sugar diet (also typically low in fruit and vegetable fibre) decreases potentially beneficial Firmicutes (such as the *Roseburia*/*Eubacterium* group and *Faecalibacterium* spp. fermenting dietary plant polysaccharides to beneficial SCFAs) and promotes increased levels of bacteria from within the Proteobacteria phylum [including mucosa‐associated enteric gut pathogens and pathobionts, such as adherent, invasive *E. coli* (AIEC) seen in increased numbers in IBD].

Short‐term dietary changes were thought to have only modest transient effects unless they are quite severe, e.g. severe energy restriction (>35% for 6 weeks), however recent evidence points to major effects following short‐term consumption of diets that are exclusively animal‐ or plant‐based, with animal‐based diets increasing the abundance of bile‐tolerant bacteria (including *Bacteroides*,* Bilophila* and AIEC) and reducing the Firmicutes metabolising dietary carbohydrates/fibre. In humans ingesting high‐protein, carbohydrate‐restricted ‘weight‐loss’ diets, weight loss is accompanied by increase in abundance of Bacteroidetes, and reduction in the *Roseburia*/*Eubacterium* group of Firmicutes. Consequently, these diets are associated with a significant reduction in the proportion of butyrate in faecal and colonic SCFA concentrations within 4 weeks which may impact on available energy resource for colonocytes. Long‐term adherence to such diets may increase risk of colonic disease. Specific carbohydrate exclusion of FODMAPs however, whilst providing gastrointestinal symptom relief for patients with IBS and increasing microbiota diversity, appears to lower relative abundance of key SCFA‐producing bacteria, e.g. *Clostridium* cluster *XIVa*.

Intervention with prebiotics (dietary carbohydrate/fibre components that encourage the growth of ‘healthy’ bacteria), particularly fructo‐ and GOS, appear to promote increased abundance of Bifidobacteria within the intestinal microbiota. This bacterial genus is known to be more prevalent in the faeces of breast milk‐fed than formula milk‐fed infants, Bifodogenesis being promoted by prebiotic human milk oligosaccharides. *In vivo* animal and human studies also provide convincing evidence to suggest that fermentable RS can also selectively promote growth of specific beneficial bacterial populations, thereby improving intestinal health. However, volunteer studies and recent clinical trials, indicate that prebiotic responses show a high‐degree of subject to subject variability, and they are also influenced by the initial composition of an individual's microbiota. More detailed analysis of the microbiota following dietary prebiotic supplementation is clearly needed.

Soluble dietary fibres (NSP), if not yet defined as impacting on the microbiome, are able to block bacteria–intestinal epithelial interactions of a range of enteric pathogens, including IBD mucosa‐associated AIEC. Not all soluble plant fibres are equally effective, with acidic (pectic) NSP from plantain (bananas) and broccoli having so far proved particularly effective, and although addition of plantain fibre to the feed substantially reduced invasion by *Salmonella* in the chicken, further studies are clearly needed to evaluate any benefit in humans. It is worth noting though, that a ‘contrabiotic’ effect is a plausible explanation for the recent demonstration from the Nurses’ Health study that subjects in the highest quintile for fruit fibre intake had ~50% lower risk for subsequent development of Crohn's disease. A combination of all these mechanisms effected by dietary fibre (insoluble and soluble components) likely explain many of the differences in microbiota associated with long‐term ingestion of a diet rich in fruit and vegetable fibre.

**Figure 5:**
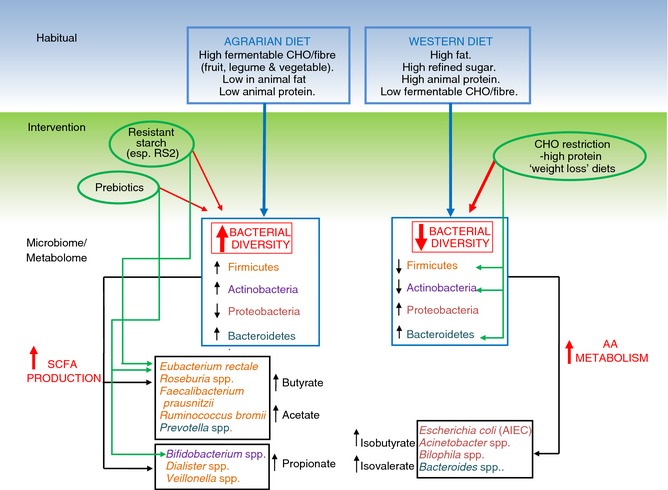
Overview of the long‐term and short‐term impact of dietary fibre on the intestinal microbiome and metabolome. An agrarian diet increases faecal microbial diversity (increased Firmicutes, reduced Proteobacteria) and encourages growth of bacteria that produce short‐chain fatty acids (such as butyrate, acetate and proprionate) ‐ all considered to be “good” for gut health. Western diet and high protein/low fermentable carbohydrate/fibre diets induce largely opposite changes which are theoretically “bad” for gut health. AA, aminoacid; AIEC, adherent, invasive *E. coli*; CHO, carbohydrate; FODMAP, fermentable oligo‐, di‐ and monosaccharides and polyols; SCFA, short‐chain fatty acids; spp., species.

An overview of the long‐term and short‐term impact of dietary fibre on the intestinal microbiome and metabolome has been presented in Figure 5.

## Authorship

*Guarantor of the article*: Barry J. Campbell.

*Author contributions*: HLS and BJC both wrote and edited the article. Both authors approved the final version of the manuscript.

*Declaration of funding interests*: BJC has received honoraria from Amgen Inc. and the Falk Foundation. Received grant/research support from BBSRC (BB/I016783/1 and BB/G01969X/1), The Wellcome Trust (074949/Z/04/Z), Provexis plc, the Bo and Vera Ax:son Johnson Foundation for Nature Medicine Limited and Arcis Biotechnology.
